# Neonatal lethality and recycling defect of transferrin receptor in mice with *Syntaxin12*/*13* disruption

**DOI:** 10.1007/s13238-018-0519-6

**Published:** 2018-03-06

**Authors:** Fang Li, Chun-Feng Liu, Yin-Zhen Xu, Yi-Lin Guo, Shu-Wen Xue, Xiang-Dong Kong, Hai-bing Zhang, Yan Zhang, Jian-Sheng Kang

**Affiliations:** 1 CAS Key Laboratory of Nutrition and Metabolism, Institute for Nutritional Sciences, Shanghai Institutes for Biological Sciences, Chinese Academy of Sciences, Shanghai, 200031 China; 2 University of Chinese Academy of Sciences, Beijing, 100049 China; 3 The First Affiliated Hospital of Zhengzhou University, Zhengzhou, 450052 China; 4 Technical Center for Animal, Plant and Food Inspection and Quarantine, Shanghai Entry-Exit Inspection and Quarantine Bureau, Shanghai, 20013 China

**Dear Editor,**

Iron deficiency, documented by World Health Organization (WHO), is the most common nutritional deficiency, and accounts for ~50% of anemia globally. Iron-deficiency anemia is notably and frequently associated with chronic heart failure, chronic kidney disease, cancer and inflammatory bowel disease. According to WHO Global Health Estimates 2014 Summary, iron-deficiency anemia is a major and prevalent public health problem worldwide, which contributes to 0.2% mortality, especially maternal and child mortality.

In adults, the human body stores approximately 3–5 g of iron. Iron in the circulation and in extravascular fluid remains nonreactive by binding with transferrin, which delivers ferric ion (Fe^3+^) to transferrin receptors (TFR) located on cell membranes. One TFR together with four transferrin-bound Fe^3+^ (two per transferrin) is internalized into endocytotic vesicle, where Fe^3+^ is released from transferrin at low pH (~5.6–6.2) and reduced by metalloreductase STEAP (six transmembrane epithelial antigen of the prostate) proteins to ferrous ion (Fe^2+^). Fe^2+^ can be transported out of endosomes via the divalent metal transporter 1 (DMT1) and the iron-depleted endosomes recycle TFR back to plasma membrane (Andrews, 2008). Cytosol Fe^2+^ is tightly constricted due to the deleterious effects of superoxide formation, which can further lead to Haber–Weiss–Fenton reaction generating the most toxic oxidant, hydroxyl radical (∙OH), in cells (Aisen, 2001). Thus, cytosol Fe^2+^ is likely protected in endosomal compartments. There are increasing evidences to support the roles of vesicular trafficking in intracellular iron homeostasis, such as exocyst complex component 6 (EXOC6, also known as Sec15l1) for the recycling of transferrin and the release of TFR exocytic vesicles (Lim et al., 2005), and sorting nexin 3 (SNX3) for the recycling of TFR and iron assimilation (Chen et al., 2013). Beyond these animal models, evidences from confocal-living imaging of reticulocytes show that TFR-containing vesicles colocalize transiently with mitochondria and endosomal iron is released into mitochondrial compartments (Sheftel et al., 2007). However, the molecules mediating the fusion events of TFR recycling still remain to be identified.

Soluble NSF attachment protein receptor (SNARE) proteins are proposed to be responsible for intracellular membrane specific fusion (Südhof and Rothman, 2009). In the present study, we identify that a Qa-SNARE, Syntaxin 12/13 (STX-T, T stands for twelve and thirteen), involves in intracellular metal homeostasis. We demonstrate that STX-T plays an essential role in the fast recycling of transferrin receptor and STX-T-deficiency mice are lethal with iron deficiency anemia.

Vesicle fusion is a key step of intracellular endosomes trafficking, and the syntaxins are known as essential components of SNARE complexs for regulated exocytosis, especially synaptic exocytosis (Südhof and Rothman, 2009). In human genome, there are 11 genes coding Qa-SNAREs (Fig. S1) out of 35 genes coding SNAREs (Bock et al., 2001). Among these Qa-SNAREs, syntaxin-1, syntaxin-2 and syntaxin-4 reside predominantly at plasma membrane, while syntaxin-5 and STX-T locate in the Golgi apparatus (Hong, 2005) and endosomes (Tang et al., 1998), respectively. It is well known that syntaxin-1 is important for synaptic vesicle exocytosis (Südhof and Rothman, 2009). On the other hand, syntaxin-2 has inhibitory role in insulin granule exocytosis (Zhu et al., 2017). However, the function of STX-T remains unknown.

To study the physiologic function of STX-T, we have silenced the expression of STX-T by inserting *GFP* gene with stop codon at the site 10 bp before the ATG start codon of *Stx-t* gene (Fig. S2). Heterozygotes (*Stx-t*^+/−^) and wild-type mice are indistinguishable, but STX-T-deficiency mice show smaller body size (Fig. 1A) and dramatically decreased body weights compared with the weights of wild-type mice at both embryonic days (E) 18.5 and postnatal day (P) 0 (Fig. 1B). Western blot analysis with the antibody against STX-T confirms the silence of STX-T expression in homozygous (*Stx-t*^−/−^) mice (Fig. 1C). None of STX-T-deficiency mice can survive beyond postnatal 12 hours (Fig. 1D).

**Figure 1:**
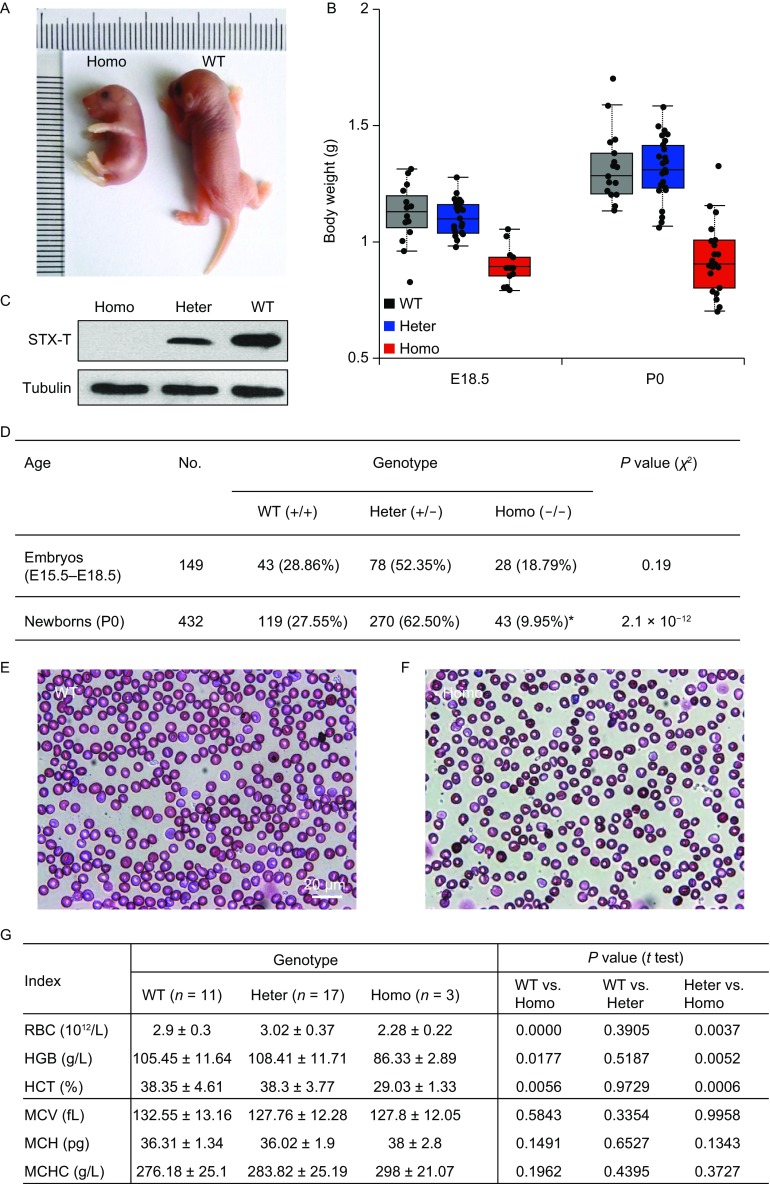
**Mice lacking** ***Stx-t*** **are lethal in postnatal 12 h**. (A) P0 littermates. Representative *Stx-t*^−/−^ mouse (Homo) on left, and wild-type mouse (WT) on right. *Stx-t*^−/−^ pup exhibits small size and stiff body compared to *Stx-t*^+/+^ littermate. (B) Scatterplots with boxplots show that *Stx-t*^−/−^ pups (Homo) have decreased weights (in g, mean ± s.d., *t* test) compared to wild-types (WT) or heterozygotes (Heter) both at E18.5 (Homo, *n* = 16, 0.95 ± 0.08; Heter, *n* = 27, 1.16 ± 0.07, *P* = 4.3 × 10^−10^; WT, *n* = 13, 1.17 ± 0.12, *P* = 5.7 × 10^−6^) and P0 (Homo, *n* = 17, 0.99 ± 0.14; Heter, *n* = 32, 1.35 ± 0.12, *P* = 5.3 × 10^*−*14^; WT, *n* = 22, 1.36 ± 0.15, *P* = 1.4 × 10^*−*9^). (C) Immunoblot analysis of STX-T. Protein samples prepared from P0 mice are blotted with antibodies against STX-T (top panel) or loading control tubulin (bottom panel). (D) The genotypic distribution of heterozygous offspring. Data are collected at embryonic and postnatal stages. Note that the genotypic distribution of wild-type (WT), heterozygous (Heter) and homozygous (Homo) littermates are analyzed and comparable to the expected numbers based on 1:2:1 ratio of Mendelian inheritance at embryonic (*P* = 0.19, *χ*^2^) and P0 stages (*P* = 2.1 × 10^*−*12^, *χ*^2^). Moreover, none of *Stx-t*^−/−^ homozygous pups (*, *n* = 43) can survive beyond postnatal 12 h. (E and F) Blood smears of wild-types (WT) and *Stx-t*^−/−^ mice, respectively. The scale bar represents 20 μm. (G) Haematological indices and statistic results of wild-types (WT), *Stx-t*^+/−^ heterozygotes (Heter) and *Stx-t*^−/−^ homozygous mice (Homo). Abbreviations: RBC, red blood cell; HGB, hemoglobin; Hct, hematocrit; MCV, mean corpuscular volume; MCH, mean corpuscular hemoglobin; MCHC, mean corpuscular hemoglobin concentration. Data represent mean ± SD

We have observed that *Stx-t*^−/−^ homozygous mice are relatively pale. Consequently, we examine the hematological indices of wild-types, heterozygotes (*Stx-t*^+/−^) and homozygous (*Stx-t*^−/−^) mice. STX-T-deficiency mice show fewer red blood cells in blood smear tests (Fig. 1E and 1F), which is further confirmed by whole blood cell analysis (Fig. 1G). Heterozygotes (*Stx-t*^+/−^) have normal hematological indices as wild-types (Fig. 1G).

Besides fewer red cells, STX-T-deficiency mice also show that hematocrit and hemoglobin values are dramatically decreased (Fig. 1G), while hemoglobin (in g/L) is decreased in STX-T-deficiency mice by 18% (86.33 ± 11.64) compared with wild type (105.45 ± 11.64). The results demonstrate that STX-T-deficiency mice suffer from iron deficiency anemia.

STX-T has been reported to be colocalized with TFR in endosomes (Prekeris et al., 1998) (Prekeris et al., 1999), but the role of STX-T in endosomes dynamics is unknown. Thus, to better study the role of STX-T in vesicle dynamics, mouse embryonic fibroblasts (MEFs) from E17.5 *Stx-t*^−/−^ and wild-type embryos are cultured. Localization of STX-T is examined in MEFs (Fig. 2A), H9C2 cell line and primary cultured hippocampal neuron (Fig. S3A and S3B), and found to be highly colocalized with TFR in these cells (Fig. S3C). The expressions and absences of STX-T are confirmed by immounblotting in wild-type and *Stx-t*^−/−^ MEFs (Fig. S 3D).

**Figure 2:**
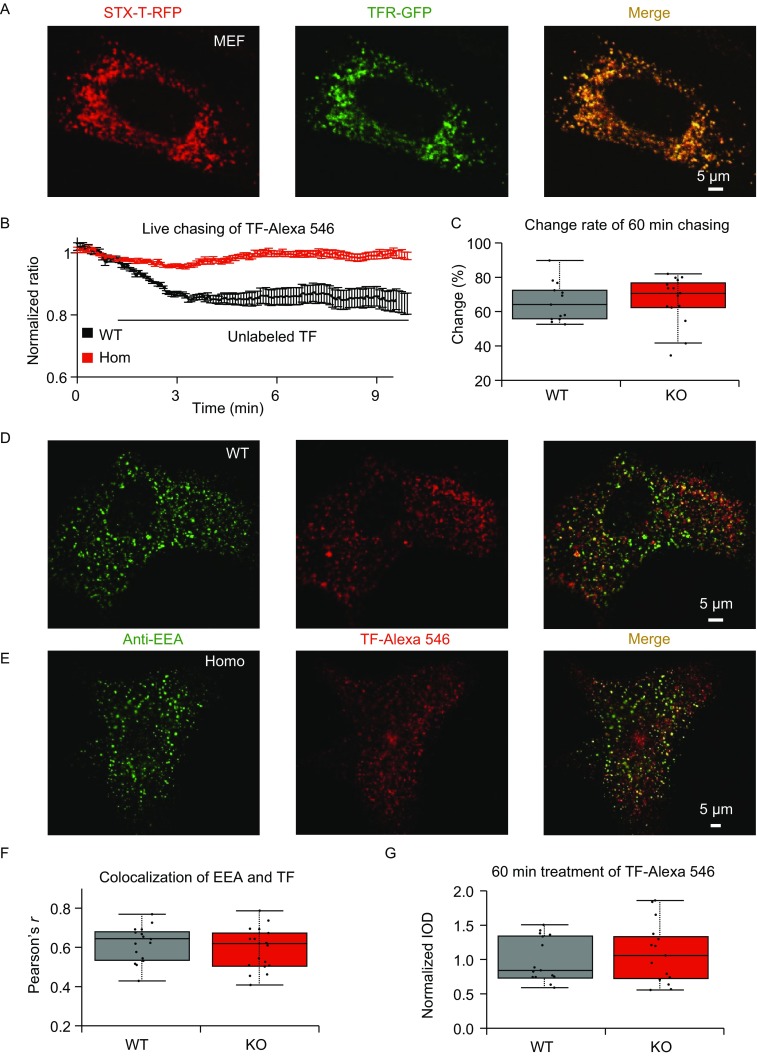
**Fast recycling deficiency of transferrin receptor in** ***Stx-t***^**−/−**^ **MEF**. (A) Colocalization of STX-T (red, RFP tagged) with TFR (green, GFP tagged) in double transfected MEFs cell. The scale bar represents 5 μm. (B) Live chasing of TF-Alexa 546 in MEFs of wild-types (WT, black, *n* = 40) and homozygotes (Homo, red, *n* = 43) shows deficiency of transferrin receptor recycling in *Stx-t*^−/−^ MEF. Error bars represent mean ± SEM. (C) Transferrin receptor recycling in *Stx-t*^−/−^ MEF (KO, *n* = 15) is normal compared to wild-types (WT, *n* = 13) for long time (60 min) chasing. (D and E) show EEA and TF-Alexa 546 in wild-type MEF (WT) and *Stx-t*^−/−^ MEF (Homo), respectively. The scale bars represent 5 μm. (F and G) Show that Alexa 546-labelled transferrin (TF, red) in *Stx-t*^−/−^ MEF (KO, *n* = 18) has normal colocalization coefficience (F, *P* = 0.44, *t* test, WT, *n* = 17) with early endosome marker (EEA, green), and comparable intensity distribution of intracellular TF-Alexa 546 (G, *P* = 0.50, *t* test, KO, *n* = 15) with wild-types (WT, *n* = 15)

To investigate the effects of STX-T on TFR recycling, MEFs labeled with Alexa-546-conjugated transferrin (TF-546) are live chasing with unlabeled TF (Fig. 2B). The fluorescence intensity of TF-546 in MEFs of *Stx-t*^−/−^ mice only show mild decay, while the intensity of TF-546 in wild-type MEFs decrease about 15% in a couple of minutes (Fig. 2B). We also did long time chasing, and found that STX-T-deficiency affect fast recycling of TFR but did not affect the long time recycling of TFR (Fig. 2C), which is very similar to the role of sorting nexin 3 (SNX3) in the recycling of TFR (Chen et al., 2013).

Furthermore, we have evaluated the effect of STX-T deficiency on TF absorption by checking the colocalization rate (Fig. 2D–G) of TF-546 and early endosome antigen 1 (EEA1) (Fig. 2D and 2E). Consequently, we have found that there are no differences about the colocalization rate and intensity of internalized TF-546 between *Stx-t*^−/−^ and wild-type MEFs (Fig. 2F and 2G). Our findings demonstrate that knockout of *Stx-t* does not affect TF-containing early endosomes process but impairs fast TFR recycling (Fig. 2B), which may lead to iron deficiency in *Stx-t*^−/−^ mice. STX-T is not necessary for the slow recycling of TFR (Fig. 2C), which could explain that STX-T-deficiency has normal absorption of TF-546. In addition, the results are consistent with the role of Qa-SNARE in fusion rather than in endocytosis.

Compared with the postnatal lethality of STX-T-deficiency mice, TFR-deficiency mice are embryonic lethal before E12.5 with anemia (Levy et al., 1999). Besides *Stx-t* and *Tfr* knockout mouse, the majority of knockout-mouse models for iron homeostasis show hereditary hemochromatosis or anemia. We have demonstrated that STX-T-deficiency mice have fewer red blood cells (Fig. 1E–G) and lower hemoglobin level (~18% decrease, Fig. 1G), which are markers of iron deficiency (Lorenz et al., 2015). The findings indicate that STX-T is necessary for erythropoiesis, which is in line with erythroid precursors are strictly dependent upon TFR-mediated endocytosis of transferrin (Levy et al., 1999). However, unlike *Tfr*^+/−^ mice showing an increase in the number of red cells (Levy et al., 1999), *Stx-t*^+/−^ mice have normal hematological indices as wild-type (Fig. 1G). In addition, unlike DMT1 which is essential for intestinal iron absorption after birth (Gunshin et al., 2005), neonatal lethality of STX-T deficiency mice suggests that STX-T has essential roles in iron homeostasis during embryonic development.

Neonatal lethality and iron deficiency suggest that loss of STX-T function can be added to the growing list of rare genetic defects causing iron-deficiency anemia (Andrews, 2008). The majority of genomic variations are attributable to single nucleotide polymorphisms (SNPs, particularly those located in protein-coding DNA regions, or cSNPs). Consequently, we have analyzed all known cSNPs and other small-scale variations (such as indels and multinucleotide polymorphisms) for the coding region of human *Stx-t* gene. A total of 90 non-synonymous cSNPs have previously been identified, some of which might affect the STX-T function (Fig. S4). More importantly, at least five frameshift and stop-gained variants are also reported, which could lead to a complete loss of STX-T function (Fig. S4). Thus, the SNP analysis of *Stx-t* gene may provide a valuable option for prenatal gene diagnosis in preventing and decreasing birth defects.

## Footnotes

We thank the following people for their help: Dr. Hong Wan Jin and Tan Yik Loo for syntaxin constructs. We thank Shanghai Research Center For Model Organisms for generating of gene targeted knockout mice. This work was partially supported by National Natural Science Foundation of China (Grant No. 31171369), the National Basic Research Program (973 Program (Nos. 2011CB910903 and 2010CB912001), and Chinese Academy of Sciences (Hundred Talents Program and 2009OHTP10).

Fang Li and Chun-Feng Liu designed and conducted the experiments and manuscript writing. Hai-bing Zhang supported Fang Li as co-mentor. Yi-Lin Guo, Shu-Wen Xue, Xiang-Dong Kong, Yin-Zhen Xu and Yan Zhang did genetic variation analysis. Jian-Sheng Kang developed the idea, directed the study and wrote the paper. All authors participated in discussions.

Fang Li, Chunfeng Liu, Yinzhen Xu, Yilin Guo, Shuwen Xue, Xiangdong Kong, Haibing Zhang, Yan Zhang and Jiansheng Kang declare that they have no conflict of interest. All institutional and national guidelines for the care and use of laboratory animals were followed. This article does not contain any studies with human or animal subjects performed by the any of the authors.

## Electronic supplementary material

Below is the link to the electronic supplementary material.

Supplementary material 1 (PDF 655 kb)

## References

[CR1] Aisen P (2001). Chemistry and biology of eukaryotic iron metabolism. Int J Biochem Cell Biol.

[CR2] Andrews NC (2008). Forging a field: the golden age of iron biology. Blood.

[CR3] Bock JB, Matern HT, Peden AA, Scheller RH (2001). A genomic perspective on membrane compartment organization. Nature.

[CR4] Chen C, Garcia-Santos D, Ishikawa Y (2013). Snx3 regulates recycling of the transferrin receptor and iron assimilation. Cell Metab.

[CR5] Gunshin H, Fujiwara Y, Custodio AO (2005). Slc11a2 is required for intestinal iron absorption and erythropoiesis but dispensable in placenta and liver. J Clin Invest.

[CR6] Hong W (2005). SNAREs and traffic. Biochim Biophys Acta BBA.

[CR7] Levy JE, Jin O, Fujiwara Y (1999). Transferrin receptor is necessary for development of erythrocytes and the nervous system. Nat Genet.

[CR8] Lim JE, Jin O, Bennett C (2005). A mutation in Sec15l1 causes anemia in hemoglobin deficit (hbd) mice. Nat Genet.

[CR9] Lorenz L, Arand J, Büchner K (2015). Reticulocyte haemoglobin content as a marker of iron deficiency. Arch Dis Child.

[CR10] Prekeris R, Klumperman J, Chen YA, Scheller RH (1998). Syntaxin 13 mediates cycling of plasma membrane proteins via tubulovesicular recycling endosomes. J Cell Biol.

[CR11] Prekeris R, Foletti DL, Scheller RH (1999). Dynamics of tubulovesicular recycling endosomes in hippocampal neurons. J Neurosci.

[CR12] Sheftel AD, Zhang A-S, Brown C (2007). Direct interorganellar transfer of iron from endosome to mitochondrion. Blood.

[CR13] Südhof TC, Rothman JE (2009). Membrane fusion: grappling with SNARE and SM proteins. Science.

[CR14] Tang BL, Tan AEH, Lim LK (1998). Syntaxin 12, a member of the syntaxin family localized to the endosome. J Biol Chem.

[CR15] Zhu D, Xie L, Kang Y (2017). Syntaxin 2 acts as inhibitory SNARE for insulin granule exocytosis. Diabetes.

