# Supplementary material for: Neonatal lethality and recycling defect of transferrin receptor in mice with Syntaxin12/13 disruption
Source: Protein Cell. 2018 Mar 6;10(1):67–71. doi: 10.1007/s13238-018-0519-6 (PMC6321817; doi:10.1007/s13238-018-0519-6)
Supplement: Supplementary file 1 — Supplementary material 1 (PDF 655 kb) [file 13238_2018_519_MOESM1_ESM.pdf]

1. Supplemental Figures and Supplemental Figure Legends

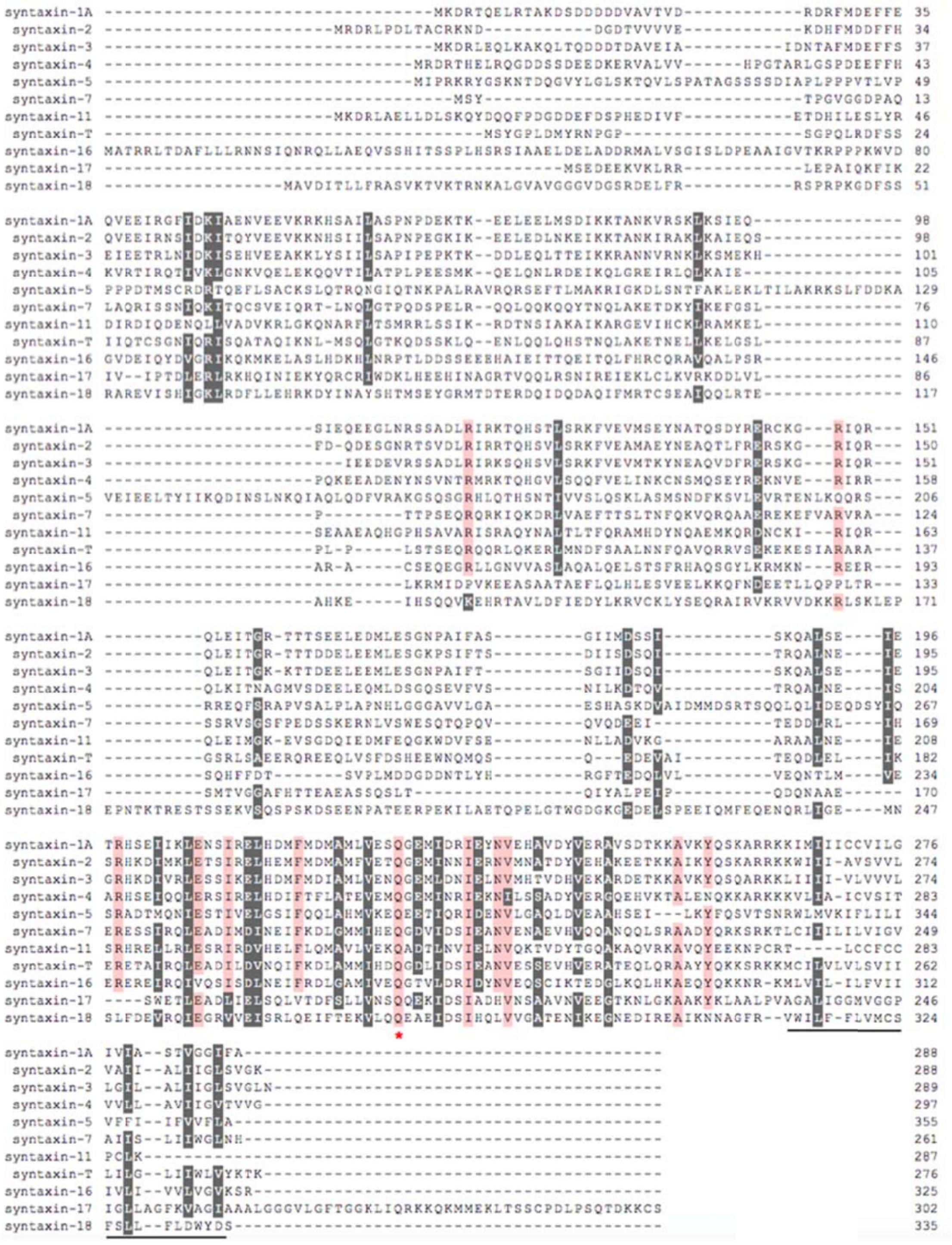

Supplementary Figure 1. Multiple alignment of 11 Qa-SNAREs. Syntaxin-T stands for syntaxin 12/13. Amino acids with more than 80% similarity are highlighted. The backgrounds of similar amino acids are dark gray, while the backgrounds of identical amino acids are light red. A red star indicates the conserved glutamine residues in helix bundle region for fusion complex forming. The C-terminal transmembrane domains are underlined. GenBank accession numbers for the human Qa-SNARE family are as follows: syntaxin-1A , NP_004594.1; syntaxin-2, NP_919337.1; syntaxin-3, NP_004168.1; syntaxin-4, NP_004595.2; syntaxin-5, NP_003155.2; syntaxin-7, NP_003560.2; syntaxin-11, NP_003755.2; syntaxin-T, NP_803173.1; syntaxin-16, NP_001001433.1; syntaxin-17, NP_060389.2; syntaxin-18, NP_058626.1.

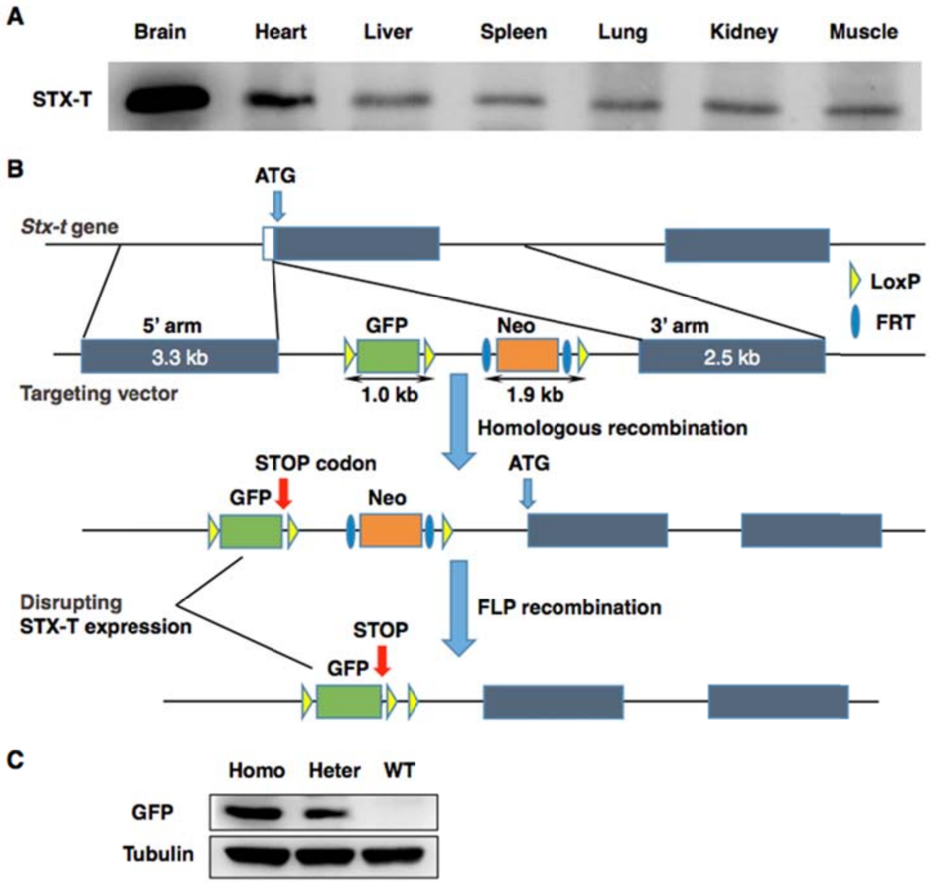

Supplementary Figure 2. Generation of *Stx-t* knockout mouse and STX-T expression level in tissues. (**A**), Immunoblot analysis of STX-T expression in E18.5 mouse tissues. Protein samples prepared from E18.5 mice are blotted with antibodies against STX-T. STX-T expresses ubiquitously and highly in brain and heart. (**B**), Schematic of generating *Stx-t* knockout mouse. To disrupt STX-T expression, the GFP gene with stop codon is inserted at the 10 bp upstream side of ATG start codon. Targeting vector is used for homologous recombination in embryonic stem cells to generate *Stx-t* knockout mice, which are further intercrossed with FLP mice to remove Neo gene. Neo, Neomycin resistance gene; FRT, flippase recognition target; FLP, flippase; LoxP, locus of X-over

P1.(**C**), Immunoblot analysis of GFP. Brain homogenates are prepared from P0 wildtype (WT), heterozygous (Heter) and homozygous (Homo) mice, and sequentially blotted with antibodies against GFP (top panel) and loading control tubulin (bottom panel).

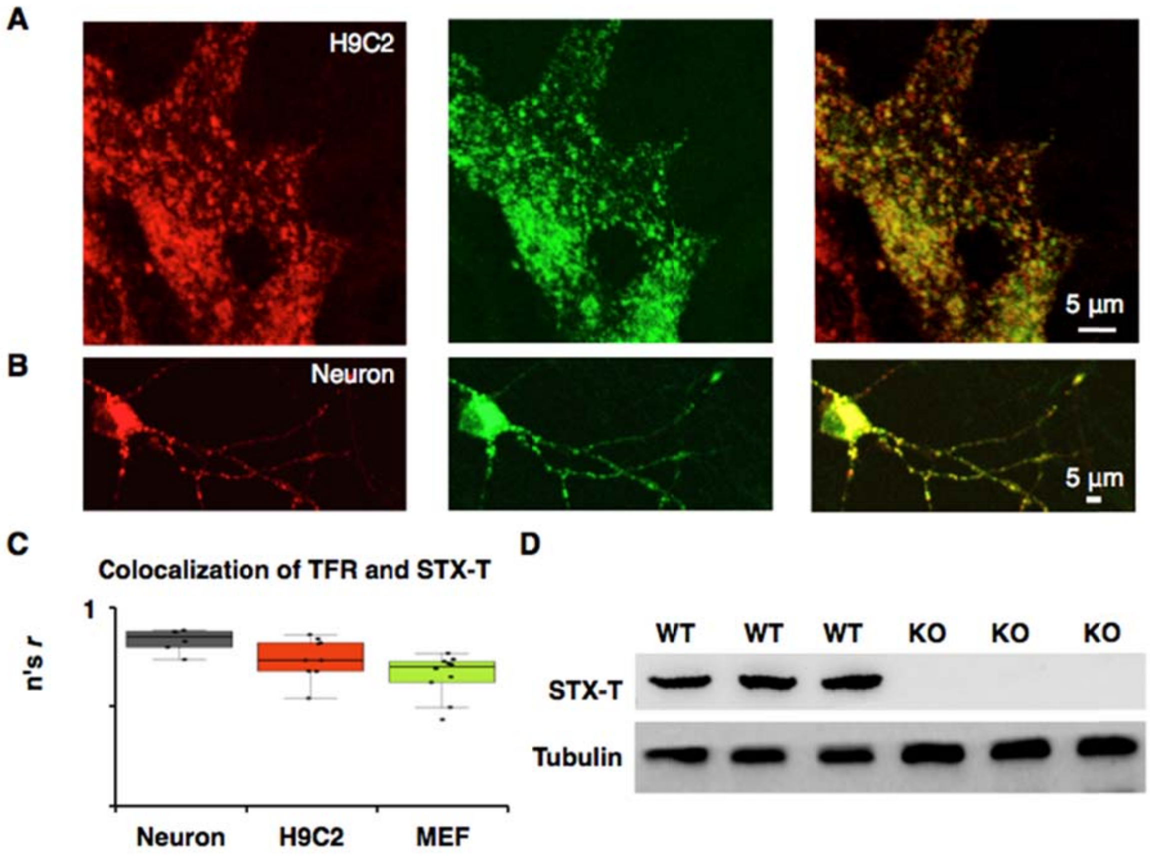

Supplementary Figure 3. Colocalization of STX-T (red, RFP tagged) with TFR. (A)-(C), Colocalization of STX-T (red, RFP tagged) with TFR (green, GFP tagged) in H9C2 (2-1) cell line (A) and primary hippocampal neuron (B) respectively. The scale bars represent 5 µm. (C), Scatterplots with boxplots show correlation coefficient (mean ±

SD) of STX-T and TFR colocalization in neuron (0.92 ± 0.03, n = 6), H9C2 (0.87 ± 0.05, n = 9) and MEF (0.83 ± 0.05, n = 10). (D), Immunoblot analysis of STX-T expressions in Mouse embryonic fibroblasts (MEFs) of wildtypes (WT) and homozygotes (Homo). Triplicates of WT and KO MEF samples are blotted with antibodies against STX-T (top) and loading control tubulin (bottom).

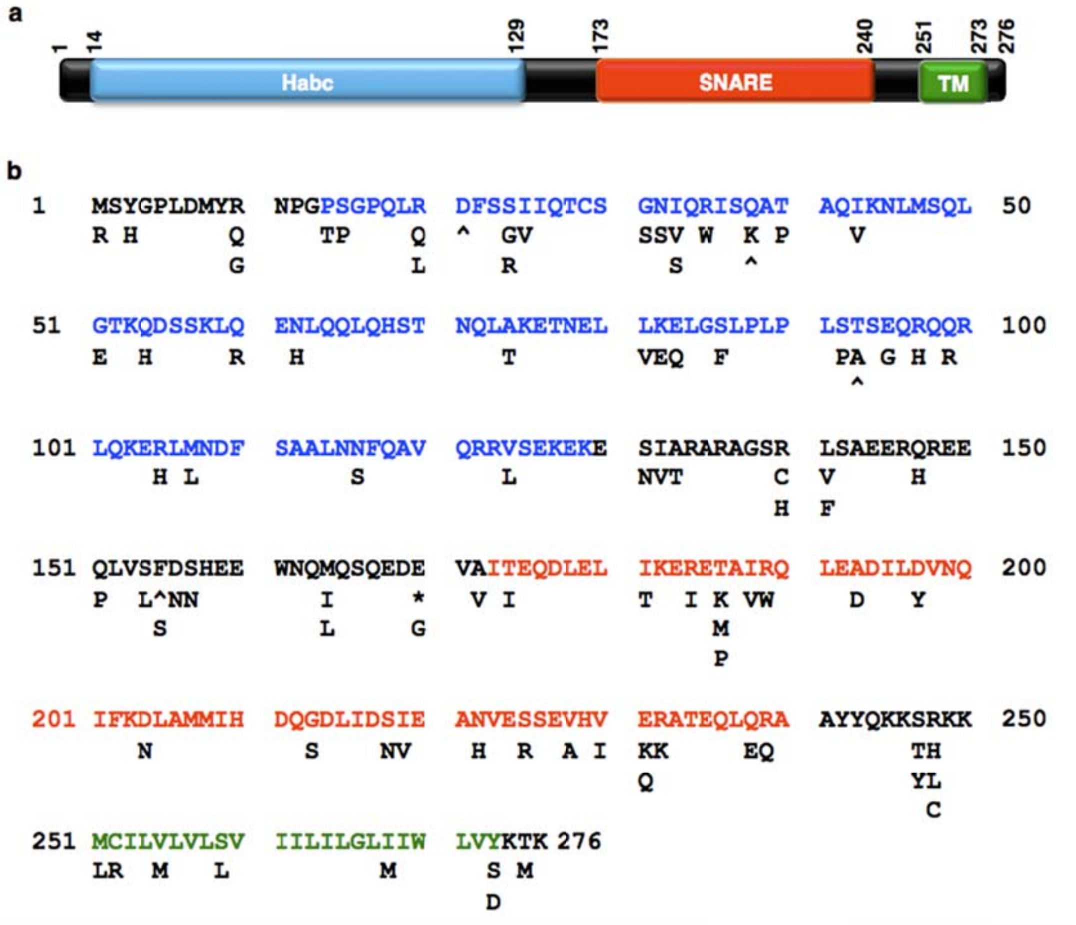

Supplementary Figure 4. Known genetic variations within the coding region of human *Stx-t* gene.(A), Human STX-T possesses a single C-terminal transmembrane domain (TM, green), a SNARE domain (SNARE, red), and an N-terminal regulatory

domain (Habc, blue). (B), The non-synonymous cSNPs and small-scale variations of STX-T. A total of 90 non-synonymous cSNPs of STX-T are shown under reference amino-acid residues, and synonymous cSNPs of STX-T are not shown. The star (*) symbol represents stop-gained variant, and caret (^) marks represent frameshit variants.

2. Materials and methods

Generation of *Stx-t* knockout mice

The *Stx-t* disruption vector contains a gene sequence *loxp-GFP-PolyA-loxp* which was inserted into the site between downstream of *Stx-t* promotor and the 10 bp upstream of start codon. The sequence of *GFP* has a stop codon and neomycin resistance gene followed by *frt* site. The targeting construct was electroporated into embryonic stem (ES) cells to replace the targeted genome sequence by homologous recombination. ES cells transfected after 24 hours were cultured with the medium containing 300 mg/L of G418 and 2 μM of Ganciclovir. ES colonies, resistant to G418 and Ganciclovir, were picked, cultured, analyzed for correct integration and confirmed by PCR analysis and sequencing. Chimeras were generated from ES clones by blastocyte injection, backcrossed with C57BL/6J, and heterozygous *GFP* knockin offsprings were confirmed by PCR analysis and sequencing. Heterozygous mice containing the *GFP* knockin sequence were intercrossed to generate *Stx-t* knockout mice. The day detecting vaginal plug is designated as embryonic day 0.5 (E0.5) and all born and alive mice to be dissected have been anesthetized by isoflurane. All experiments were conducted in accordance with the guidelines of the Institutional Animal Care and Use Committee of the Institute for Nutritional Sciences, Shanghai Institute for Biological Sciences, Chinese Academy of Sciences.

Isolation of Mouse Embryonic Fibroblasts (MEFs)

Sacrifice anesthetized pregnant mouse at embryonic 17.5 and dissect out the uterine, immersing into 75%(v/v) ethanol and rinsing with PBS without Ca^{2+} and Mg^{2+} (Gibco, Invitrogen). Separate each embryo from its placenta, remove head, limbs and viscera and mince the remaining tissue into pieces as small as possible. Use part of tail for genotype identification. Digest the tissues in fresh 15 ml 0.05% trypsin for 30 min at 37 °C, and shake the 50 ml tube every 10 min. Inactivate the trypsin by adding same volume of MEF medium (10 ml FBS plus 90 ml DMEM), centrifuge cell at 1000 g for 5 min and then remove the supernatant, repeat once time; Add 10 ml of MEF medium and transfer to a 55 cm^2 Petri dish for incubation.

The internalization and recycling of transferrin in MEFs

After incubating with 25 μg/ml conjugated with Alexa Fluor-546 for 30 min or 60 min, MEF cells were live chased with 100 μg/ml non-fluorescent holo-TF (Calbiochem, Cat No: 616420), the living imaging were recorded for about 10 min with the customized fluorescence microscope with Evolve-512 EMCCD (Photometrics Ltd., USA) as camera and Optoscan monochromator (Cairn Research Ltd., UK) as light source, and images were taken every 5 seconds. The images were analyzed with ImageJ plugins “Intensity v Time monitor of Stack-T function” and the fluorescence intensity was normalized with the mean value of first 15 images before adding holo-TF.

Statistical analysis

Data are pooled from or repeated for each condition at least three independent experiments. No statistical methods were used to predetermine sample size. Data of live chasing in Fig. 2B are represented with mean values and the standard error of mean (SEM) (Mean ± SEM), All data in text or figures are indicated with mean values and the corresponding standard deviation (SD) (Mean ± SD). The genotypic distribution of heterozygous offspring is analyzed using Chi-squared test (χ^2), and other results were analyzed statistically by two tailed Student *t-test*. When the value *P* is less than 0.05, differences are considered significant.

Westernblot

Mouse tissues were homogenized in cold lysis buffer (1% Triton X-100 and 1% DOC in Tris-buffered saline buffer) with phosphatase and protease inhibitors using micro tissue grinders (Kimble 749540-0000, USA), incubated on the ice for 30 min to lysis cell completely, while MEF cells could directly be grinded, centrifuged at 12,000 rpm for 15 min at 4 °C, the supernatants (protein lysates) were collected and its concentration were determined by BCA protein assay kit (CWBIO, Cat NO: CW0014). Extracted proteins were separated using 4~20% gradient gel electrophoresis and electrotransferred to Protran Nitrocellulose membranes (PerkinElmer) with a constant voltage of 120 for 1.5 hours. The transferred membranes were blocked by 5% nonfat dry milk in Tris-buffered saline with 0.05% Tween 20 for 1 hour at room temperature, incubated with first antibody for overnight at 4 °C with gentle shaking, washed in Tris-buffered saline with

0.05% Tween three times (10 minutes per time), finally incubated for 1 hour with anti-rabbit (GE, 1:2000, Cat No: NA934) or anti-mouse (Cell Signaling, 1:2000, Cat No: 7076S) second antibody at room temperature and washed three times again in Tris-buffered saline with 0.05% Tween. First antibodies were used recognizing Syntaxin12/13 (Abcam, 1:1000, Cat No: ab13261), β-Tubulin (Sigma, 1:2000, Cat No: T4026), GFP (Abmart, 1:2000, Cat No: P30010), α-Actin (Abmart, Cat No: M20010) The immunoblots were visualized with ECL Western Blotting Substrate (Pierce, Cat No: 32109) and exposed the blots to imager (GE, ImageQuant LAS 4000 mini).

Plasmid construction, cell culture and transient transfection

The DNA fragments encoding TFR and STX-T were amplified from rat brain genomic complementary DNA (cDNA) and inserted into the expressing vector pEGFP-N1 Vector (Invitrogen) and TagRFP-T-N1 vector respectively. Primary hippocampal neuron culture process was described previously(Kang et al., 2008). H9C2 (2-1) cell lines obtained from the Cell Bank of the Chinese Academy of Sciences (Shanghai, China) was cultured in DMEM with 10%FBS at 37 °C in 95% air and 5% CO_2. Cells were seeded at a density of $1x10^4$ cells per cm^2 and then transfected transiently by calcium-phosphate method. TFR-GFP construct was co-transfected with STX12-RFP vector. After transfection, cells were cultured for additional two days and imaged with confocal microscope (FV1000; Olympus), the colocalization between TFR-GFP and STX12-RFP was analyzed with imageJ plugin “colocalization Indices” (by Kouichi Nakamura, Kyoto University) and represented with correlation coefficient.

Whole blood collection and analysis

Blood smears were made by putting a small drop of blood on a glass slide and then spreading in a thin film over the slide using the edge of another slide. Air-dry blood smears were stained with Wright-Giemsa stain (Solarbio, Cat No: 1020) according to the manufacturer's manual. A volume of 20 µl whole bloods were collected by using spray-coated EDTA•K_2 collection tubes from decapitated E18.5 embryos, diluted to final volume 200 µl, and assayed in XT 2000i Automated Hematology Analyzer (Sysmex).

Immunostaining

MEF cells seeded on glass coverslips were starved for 2 hours in DMEM medium without serum, placed on ice for 10 min and then incubated with 25 µg/ml Transferrin (TF) conjugated with Alexa Fluor 546 (Invitrogen, Cat No: T23364), being washed one time with PBS and fixation for 30 min at room temperature (RT) in 4% FSB solution containing 4% formaldehyde (Polyscience Inc.), 4% sucrose (Sigma-Aldrich) in 1x PBS, cell were rinsed three times with 1x PBS, permeabilized and blocked with 0.5% Triton X-100 and 5% serum in 1X PBS for 30 min at RT, incubated with early endosome antigen 1 (EEA1) antibodies (BD, 1:200, Cat No: 610456) over night at 4 °C, rinsed three times with 1x PBS, incubated with Alexa 488 F(AB') goat anti-mouse secondary antibody (Invitrogen, 1:400, Cat No: A-10684) for 30 min at RT, imaged with confocal microscope and also analyzed the colocalization rate between TF-Alex 546 and EEA1.

SNP analysis

Known single nucleotide polymorphisms (SNPs) and small-scale variations of *Stx-t* gene were retrieved from the dbSNP database (NCBI, build 148).
